# Vhl safeguards thymic epithelial cell identity and thymopoietic capacity by constraining Hif1a activity during development

**DOI:** 10.1016/j.isci.2024.110258

**Published:** 2024-06-13

**Authors:** Christiane Grammer, Julia A. Komorowska, Jeremy B. Swann

**Affiliations:** 1 Department of Developmental Immunology, Max Planck-Institute of Immunobiology and Epigenetics, Freiburg, Germany; 2 Albert Ludwig University, Faculty of Biology, Freiburg, Germany

**Keywords:** Immunology, Molecular Biology, Molecular Genetics

## Abstract

The thymus is a physiologically hypoxic organ and fulfills its role of generating T cells under low-oxygen conditions. We have therefore investigated how thymic epithelial cells (TECs) cope with physiological hypoxia by focusing on the role of the Hif1a–Vhl axis. In most cell types, the oxygen-labile transcriptional regulator Hif1a is a central player in co-ordinating responses to low oxygen: under normoxic conditions Hif1a is rapidly degraded in a Vhl-guided manner; however, under hypoxic conditions Hif1a is stabilized and can execute its transcriptional functions. Unexpectedly, we find that, although TECs reside in a hypoxic microenvironment, they express little Hif1a protein and do not require Hif1a for their development or function. Instead, we find that Vhl function in TECs is vital to constrain Hif1a activity, as loss of Vhl results in dramatic defects in TEC differentiation and thymopoiesis, which can be rescued by Hif1a co-depletion.

## Highlights

• Vhl is required for normal development of mouse thymic epithelial cells
• Development of Vhl-deficient TECs can be largely restored by co-depletion of Hif1a
• Hif1a-deficient TECs develop normally, even in the low-O_2_ environment of the thymus

## Graphical abstract

**Figure:**
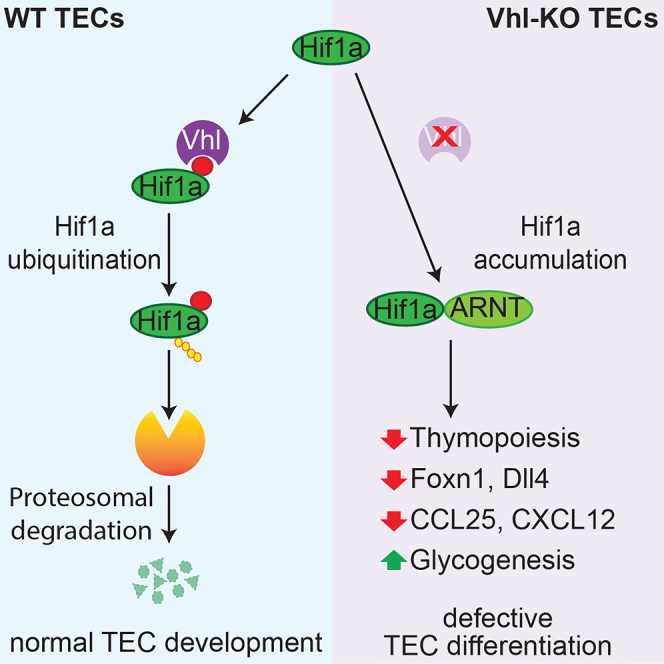

## Introduction

The thymus is an evolutionarily conserved primary lymphoid organ responsible for the production of T cells.^1^ This process, referred to as thymopoiesis, involves a complex interplay between both stromal and hematopoietic cell types, which occur in highly specialized microenvironments^2^ established by Foxn1-dependent thymic epithelial cells (TECs).^3^ Defects that interfere with the establishment of distinct thymic microenvironments can have severe consequences in the form of immunodeficiency, when T cell production fails, or autoimmunity due to a failure in central tolerance. Identifying factors that maintain the health of the thymic microenvironment is therefore key to understanding how healthy thymus function is established and maintained.

One fundamental consideration for thymus function is the adequate provision of nutrients such as oxygen. As is the case for all organs, the circulatory system is responsible for the delivery of oxygen to the thymus, and proper development of the thymic vasculature plays a key role in thymus organogenesis and microenvironment organization.^4^^,^^5^^,^^6^^,^^7^ Curiously, despite having a defined vascular network, measurements of intra-thymic oxygen concentrations have consistently revealed the thymus to be a low-oxygen environment.^8^^,^^9^ Measurements taken with fiber-optic or microelectrode probes both indicate that thymic pO_2_ averages around 10 mmHg, a value that would be considered hypoxic in most other organs. Additionally, it was noted that many measurements were substantially lower (<5 mmHg), suggesting that there may be some microenvironmental variability in oxygen levels within the thymus. These findings correlate well with the staining pattern observed when mice are treated with the hypoxia probe pimonidazole: positive foci were observed in both the thymic cortex and medulla, and staining intensity (as well as the number of stained cells) tended to correlate with distance from blood vessels. Several subsequent studies have since confirmed the hypoxic nature of the thymus under steady-state conditions.^10^^,^^11^^,^^12^

These results imply that, *in vivo*, thymopoiesis occurs in a lower-oxygen environment than is found in most other organs.^13^ In fact, the prevailing conditions in the various thymic microenvironments would be classed as mildly hypoxic to hypoxic,^14^ or ranging from physiologically to pathologically hypoxic,^15^ depending on the classification system used. Therefore, it would seem necessary that both the stromal cells that reside permanently within the thymusand the mobile thymocytes that develop in the thymus over a period of 2–4 weeks^16^ would have evolved mechanisms by which they can adapt to the local low-oxygen conditions within this organ.

Hypoxia-inducible factors (HIFs) are critical mediators of the cellular response to low-oxygen conditions in a range of organs and cell types.^17^^,^^18^ These molecules act as transcriptional regulators that alter gene expression in an oxygen-sensitive manner, and their activity is controlled primarily at the level of protein stability. HIF complexes consist of an oxygen-labile α subunit, which pairs with a stable β subunit (aryl hydrocarbon receptor nuclear translocator, ARNT) to form a DNA-binding heterodimer that in turn binds to hypoxia response elements (HREs) in the genome to modulate gene expression. These heterodimers only form under hypoxic conditions as, when oxygen is present, the HIFa subunits are continually degraded via the proteosome. This oxygen-dependent stability is regulated through enzymatic activity. In the presence of oxygen, prolyl-hydroxylase (PHD) and factor-inhibiting HIF1 (FIH1) enzymes hydroxylate HIFa proteins, which allows them to be recognized by the von Hippel-Lindau protein (Vhl). Vhl is the substrate-recognition module of an E3-ubiquitin ligase complex, which ubiquitinates hydroxylated HIFa subunits, thereby targeting them to the proteasome for destruction. When oxygen levels become limiting, the enzymatic activity of PHD and FIH1 activity is lost or reduced, and HIFa subunits are no longer targeted for degradation. Consequently, the subunits begin to accumulate and heterodimerize with HIFb. HIF heterodimers then translocate to the nucleus, where they regulate expression of a number target genes—often in a cell-type- or tissue-specific manner—to co-ordinate cellular responses to hypoxia. These transcription changes influence a number of biological processes, causing metabolic adaptations, changes in proliferation, and triggering angiogenic and erythropoietic processes to allow cells and tissues to respond to hypoxic challenges.

Given that oxygen levels within the thymus are lower than the threshold at which Hif1a expression is typically induced (protein levels are thought to begin increasing at 6% O_2_, or ≈40 mmHg,^18^), we set out to determine the role of Hif1a and Vhl in the biology of TECs. Our investigations revealed that, even though the thymic microenvironment is physiologically hypoxic, Hif1a is dispensable for thymus function. In contrast, forced induction of a Hif1a-driven transcriptional response in TECs was highly detrimental to thymus development. Inactivation of Vhl in TECs resulted in Hif1a overexpression, which blocked differentiation, and induced a glycogenic phenotype. Our results therefore reveal an unexpected outcome: despite developing in a physiologically hypoxic environment, TECs are not reliant on Hif1a activity for their function and remain dependent on Vhl to prevent Hif1a protein accumulation from impeding thymopoiesis.

## Results

### Conditional deletion of Vhl in TECs blocks both TEC and T cell differentiation

**Figure 1:**
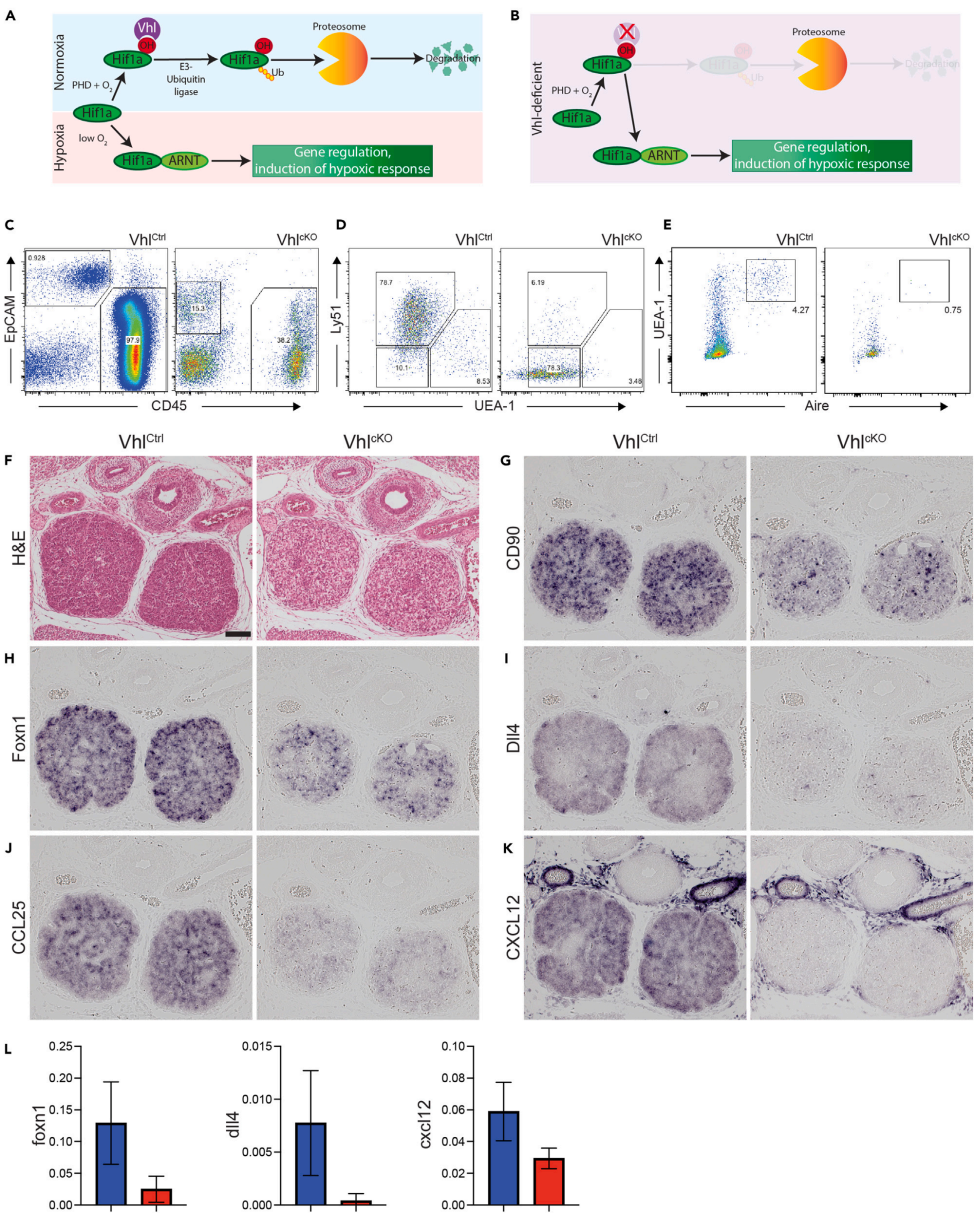
Defective TEC development in the absence of Vhl Schematics outlining the fate of Hif1a under conditions of normoxia or hypoxia (A), or under conditions of Vhl deficiency (B), are shown (see Figure S4 for more details). Staining to identify TEC and hematopoietic cell subsets in thymi from Vhl^Ctrl^ or Vhl^cKO^ E18.5 embryos is shown in (C). TECs are defined as CD45^−^EpCAM^+^; note that in the case of Vhl^cKO^ embryos EpCAM down-regulation necessitated the use of an alternative gating strategy. UEA-1 and anti-Ly51 staining of gated CD45^−^EpCAM^+^ TECs is shown in (D), and intracellular staining for Aire-expression is shown in (E). Representative gates defining cTEC and mTEC subsets are depicted; cTECs were defined as Ly51^+^, mTECs as UEA-1^+^, and unstained cells were assigned as being double negative (DN). Mature mTECs were defined as UEA-1^+^Aire^+^. Further details regarding cell counts and proportions can be found in Figures S1 and S2. H&E staining (F) and ISH (G–K) were used to examine thymopoiesis in Vhl^Ctrl^ and Vhl^cKO^ embryos at E15.5. Expression of CD90 (G), Foxn1 (H), Dll4 (I), CCL25 (J), and CXCL12 (K) was reduced in Vhl^cKO^ thymi. Each panel (F–K) shows representative staining from a control (left) and conditional knockout (right) embryo; the depicted results are representative of 3 embryos examined per genotype. The scale bar in (F) represents 100 μm and applies to all subsequent panels. (L) qPCR was used to quantify gene expression in whole thymi isolated from Vhl^Ctrl^ (blue) and Vhl^cKO^ (red) E15.5 embryos; results are from *n* = 4 biological replicates for each genotype (mean ± SD).

A schematic outlining the canonical Hif1a–Vhl axis is shown in Figure 1A, and we began our study by testing the consequences of Vhl deficiency in TECs (Figure 1B). As complete Vhl deficiency is embryonic lethal,^19^ we used a conditional-knockout approach to deplete Vhl in TECs, based upon a floxed Vhl allele,^20^ combined with a Foxn1-Cre driver^21^ which is active in TECs and skin keratinocytes. We first analyzed thymus development at embryonic day 18.5 (E18.5), the latest stage possible in this model, as it is known that Vhl deficiency in the skin can cause stunted post-natal growth and lethality.^22^^,^^23^ We compared Vhl^+/fl^; Foxn1-Cre^+^ (hereafter Vhl^Ctrl^) with Vhl^fl/fl^; Foxn1-Cre^+^ (Vhl^cKO^) littermates and found that TEC-specific Vhl deficiency resulted in a dramatic drop in thymocyte number at E18.5 (Figure S1A). Flow cytometric analysis using CD45 and epithelial cell adhesion molecule (EpCAM) markers revealed the expected hematopoietic (CD45^+^EpCAM^−^) and TEC (CD45^−^EpCAM^+^) populations in Vhl^Ctrl^ thymi, but surprisingly the typically bright-staining EpCAM^+^ TECs were absent in Vhl^cKO^ thymi, which instead contained a CD45^−^EpCAM^lo^ population (Figure 1C). Cell counts and flow cytometry showed that CD45^+^ thymocytes were reduced in Vhl^cKO^ thymi (Figure S1B), but both the proportions and total numbers of TECs (gated as depicted in Figure 1C to account for the varying EpCAM levels in each genotype) were increased (Figure S1C). To better characterize the unusual TEC population in Vhl^cKO^ mice, we next tested for markers of cortical (cTEC) and medullary (mTEC) differentiation. Co-staining with Ly51 and UEA-1 revealed the expected populations of cTECs (UEA-1^-^Ly51^+^) and mTECs (UEA-1^+^Ly51^-^) in Vhl^Ctrl^ thymi; however, Vhl^cKO^ TECs were mostly negative for both differentiation markers (Figures 1D, S1D, and S1E). Additional characterization of TEC differentiation revealed that at E18.5 mTECs expressing the autoimmune regulator (Aire) were virtually absent from Vhl^cKO^ thymi (Figures 1E and S2A) and that expression of both class I and II major histocompatibility complex (MHC) was significantly reduced in Vhl^cKO^ TECs (Figures S2B and S2C). We also performed staining for Ki67 to test if Vhl deficiency influenced TEC proliferation but found no difference in Ki67 expression at either E15.5 or E18.5 (Figure S2D). These results demonstrate that Vhl deficiency causes significant disruption in TEC differentiation, but not proliferation, and substantially compromises the capacity of the embryonic thymus to support thymopoiesis.

### Disruption of Foxn1-driven genetic network in TECs

Examination of H&E-stained sections from E15.5 Vhl-deficient thymi revealed that, although the thymic lobes were correctly positioned within the mediastinum at this time point, they had a much more open structure, contrasting with the densely packed cells present in control thymi (Figure 1F). *In situ* hybridization (ISH) using a CD90 (Thy1) probe demonstrated a substantial reduction of CD90-expressing cells in Vhl^cKO^ thymi (Figure 1G), a finding consistent with the flow cytometry data described earlier. We then used ISH to examine the expression of several TEC-expressed genes with known roles in thymopoiesis. First, we examined the expression of Foxn1 itself and found that Vhl^cKO^ thymi consistently displayed reduced Foxn1 expression (Figure 1H). The expression of Dll4 (Figure 1I), an essential factor for instructing T cell differentiation, as well as the chemokines CCL25 (Figure 1J) and CXCL12 (Figure 1K), was all reduced in Vhl^cKO^ thymi. Down-regulation of foxn1, dll4, and cxcl12 transcripts in Vhl^cKO^ thymi was confirmed by qPCR (Figure 1L), validating the reductions detected by ISH. Together, these results demonstrate that Vhl-deficient TECs fail to express several key factors essential for thymopoiesis, including the chemokines required for efficient recruitment of lymphoid progenitors,^24^^,^^25^^,^^26^ as well as the central factor required for directing progenitors toward a T cell fate.^27^^,^^28^ The reduction of Foxn1 expression is also suggestive of an early defect in TEC differentiation or stability.

### Early block in TEC differentiation

**Figure 2:**
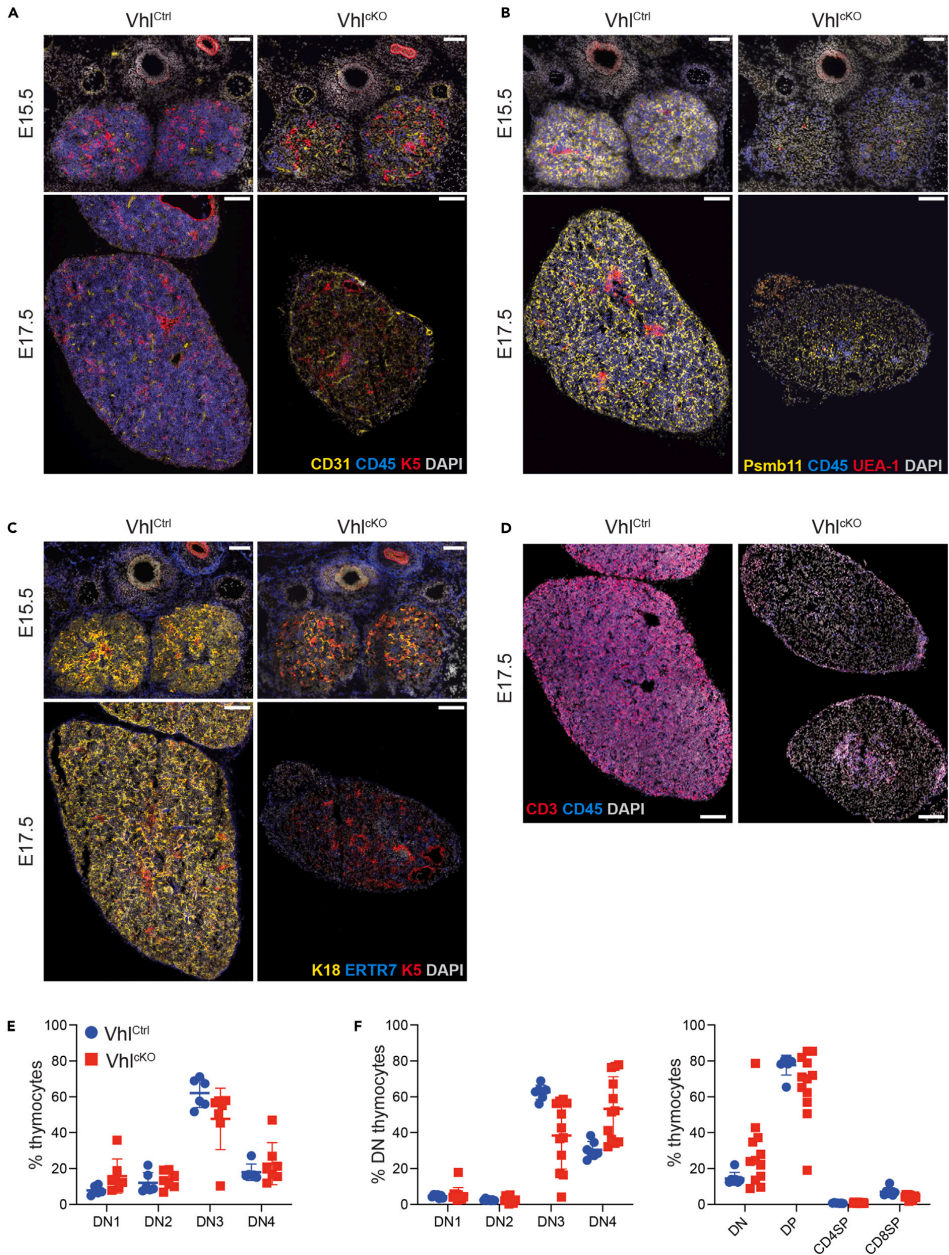
Histological defects and residual thymopoiesis in Vhl-deficient thymi Sections of thymic lobes from E15.5 and E17.5 embryos were stained for the indicated markers and evaluated by confocal microscopy. Each panel in (A–D) depicts representative sections from Vhl^Ctrl^ (left column) and Vhl^cKO^ (right column) embryos at E15.5 (top rows) or E17.5 (bottom rows). A color key identifying the specific markers used is indicated for each panel, scale bars (in white) represent 100 μm. Three embryos were analyzed for each genotype per time point. Flow cytometry was used to characterize thymocyte subsets at E15.5 (E) and E18.5 (F) time points. DN subsets were defined on the basis of CD44 and CD25 expression, and CD4 and CD8 co-staining was used to define DN, DP, CD4SP, and CD8P subsets. Each data point represents a single embryo; mean ± SD is indicated for each group.

We then used immunofluorescent (IF) staining to further characterize the thymic microenvironment in Vhl^cKO^ thymi. Consistent with the flow cytometry data, and CD90 ISH results, IF staining again confirmed a drastic reduction in the number of hematopoietic cells in the thymus of Vhl^cKO^ embryos (Figures 2A–2D). Co-staining for the endothelial marker CD31 and the mTEC marker Keratin-5 revealed that, although they were poorly populated with thymocytes, E15.5 Vhl^cKO^ thymi were vascularized and contained some K5-positive epithelial cells (Figure 2A). K5 staining is typically associated with an mTEC phenotype; however, the flow cytometry results described earlier suggested that both cTECs and mTECs were reduced in Vhl^cKO^ thymi. To reconcile this apparent discrepancy, we next investigated proteasome subunit beta type-11 (Psmb11) expression and UEA-1 staining in Vhl-deficient thymi. In the embryonic thymus Psmb11 is a marker of both progenitor and cTECs,^29^^,^^30^^,^^31^ while UEA-1 reactivity is restricted to the developing mTEC subset.^32^ Strong Psmb11 expression could be readily detected in Vhl^Ctrl^ thymi; however, staining was weak or absent in thymi from Vhl^cKO^ embryos, indicating an early-stage failure of TEC differentiation in the absence of Vhl (Figure 2B). Importantly, despite the presence of K5^+^ epithelial cells in Vhl-deficient thymi, little to no UEA-1 staining was detected in Vhl^cKO^ thymi at both E15.5 and E17.5 time points (Figure 2B), consistent with the phenotype observed by flow cytometry. We interpret these results to mean that the K5^+^ cells detected in Vhl^cKO^ thymi are unlikely to represent bona fide mTECs and instead indicate an undifferentiated TEC phenotype; Keratin-18 (K18) staining (Figure 2C) also confirmed a lack of cTEC differentiation, in agreement with both the results of Psmb11 and Ly51 staining by immunofluorescence and flow cytometry, respectively. The lack of differentiated TEC subsets correlated with a loss of thymopoietic capacity, as co-staining with CD45 and CD3 revealed the presence of abundant T-committed thymocytes in E17.5 control thymi. Remarkably, such cells were rare in Vhl^cKO^ thymic lobes (Figure 2D). To further characterize the few remaining thymocytes present in Vhl^cKO^ thymi, we examined double-negative DN1–DN4 subsets (as defined by CD44/CD25 expression) by flow cytometry and found that the proportions of DN subsets were similar in Vhl^Ctrl^ and Vhl^cKO^ thymi at E15.5 (Figure 2E). At E18.5 we detected a decrease in DN3 thymocyte proportions, and a corresponding increase in the proportion of DN4 cells; however, staining with CD4 and CD8 revealed no subsequent change in CD4^+^CD8^+^ double-positive (DP) thymocyte proportions (Figure 2F). Therefore, while the number of thymocytes is severely reduced in Vhl^cKO^ thymi, the few cells that are recruited to the thymus can still differentiate to the DP stage, albeit in very limited numbers. These results are consistent with the reduction, but not complete ablation, of essential thymopoietic factors observed in Vhl^cKO^ thymi and suggest that residual expression of these factors can support limited thymopoiesis. These results demonstrate that Vhl deficiency blocks TEC differentiation at an early stage of development, rendering these cells incapable of supporting normal thymopoiesis.

### Deregulation of the Vhl-Hif1a axis

**Figure 3:**
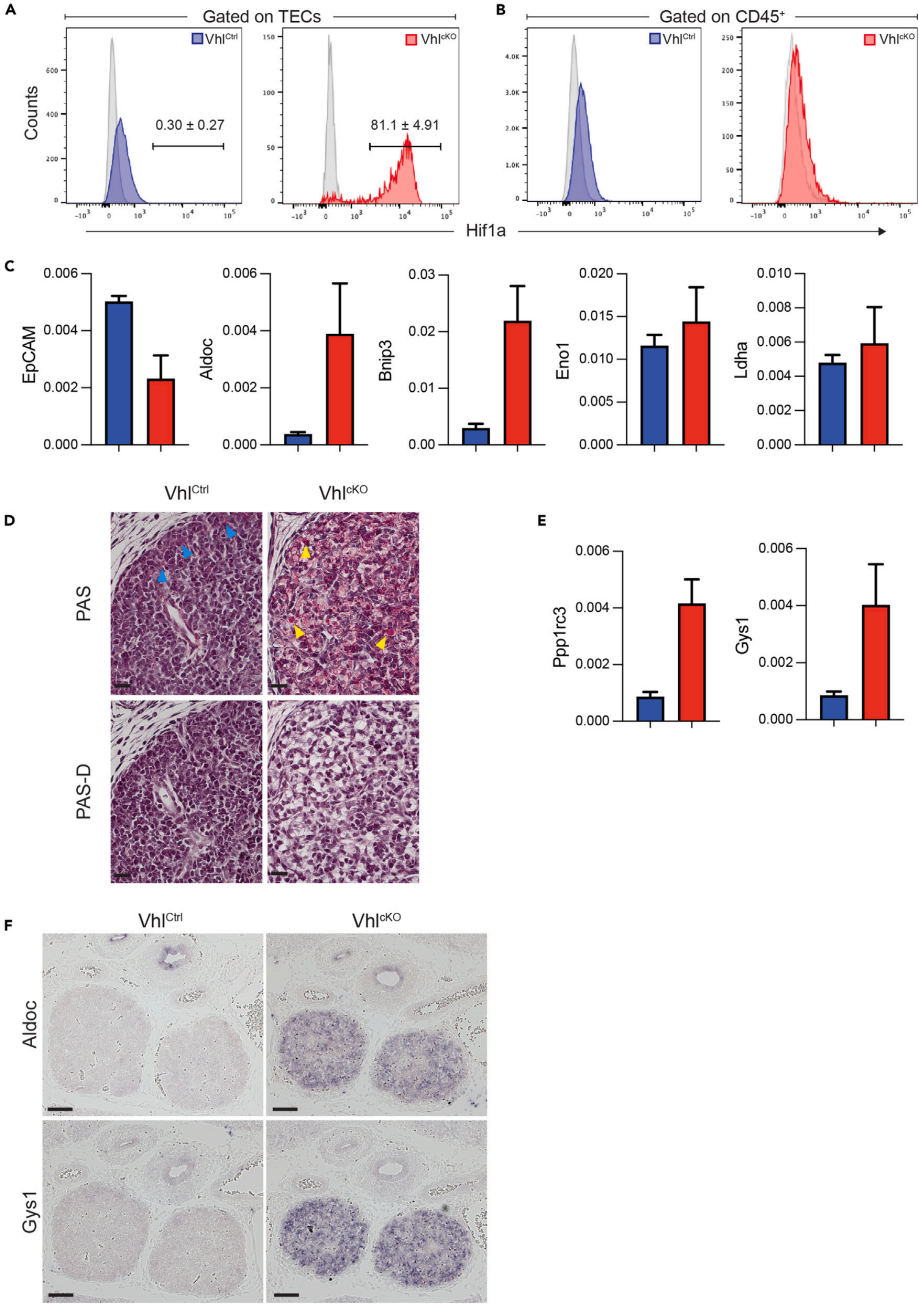
TEC-specific Vhl deficiency induces gene expression changes and glycogen storage Loss of Vhl results in a dramatic increase in Hif1a protein levels in TECs (A&B). Cell suspensions were generated from E17.5 Vhl^Ctrl^ and Vhl^cKO^ thymi, surface stained for CD45 and EpCAM, then fixed and stained intracellularly to detect Hif1a expression. Representative histograms depicting Hif1a staining of CD45^−^EpCAM^+^ TECs (A) or CD45^+^EpCAM^-^hematopoietic cells (B) are shown; blue and red overlays are from Vhl^Ctrl^ and Vhl^cKO^ samples, respectively; isotype control staining is shown in gray. Events falling in the gate depicted in (A) were designated as Hif1a^hi^, and the values shown above the gate indicate the percentage of Hif1a^hi^ TECs for each genotype (mean ± SD, *n* = 9 and 4 for Vhl^Ctrl^ and Vhl^cKO^, respectively). (C) qPCR was used to quantify gene expression in whole thymi isolated from Vhl^Ctrl^ (blue) and Vhl^cKO^ (red) E17.5 embryos. (D) Sections from E15.5 Vhl^Ctrl^ (left panels) and Vhl^cKO^ (right panels) were subjected to PAS (top row) or PAS-D (bottom row) staining protocols. Blue arrowheads indicate the fine PAS^+^ granules observed in the cortex of Vhl^Ctrl^ thymi; yellow arrowheads highlight some of the large PAS^+^ granules found in Vhl^cKO^ thymi. Note the accumulation of PAS^+^ (magenta) material in Vhl^cKO^ embryos, which is lost after dispase treatment, indicative of glycogen (see also Figure S3). (E) qPCR analysis of Ppp1rc3 and Gys1 expression in E17.5 thymi, as in (C). ISH (F) was used to confirm that Aldoc (top row) and Gys1 (bottom row) were up-regulated in E15.5 thymi from Vhl^cKO^ embryos. qPCR results in (C and E) are from *n* = 4 biological replicates for each genotype. In (D and F) scale bars indicate 100 μm, and the results are representative of at least 3 embryos examined per genotype.

To understand the mechanism by which Vhl loss impaired TEC differentiation and function, we next determined the effect of Vhl deficiency on Hif1a protein expression. Intracellular staining revealed that Hif1a protein was low or absent in control TECs but accumulated to high levels in Vhl-deficient TECs (Figure 3A). This effect was cell intrinsic, as CD45^+^ hematopoietic cells in the same samples failed to show an increase in Hif1a expression (Figure 3B). We then used qPCR to determine the consequences of Hif1a stabilization on gene expression in TECs. First, we checked expression of EpCAM and found that EpCAM mRNA levels were reduced in Vhl^cKO^ thymi (Figure 3C), consistent with what we previously observed by flow cytometry (Figure 1C). We then determined the expression levels of a set of known Hif1a target genes, Aldoc (aldolase C), Bnip3 (BCL2/adenovirus E1B interacting protein 3), Eno1 (enolase 1), and Ldha (lactate dehydrogenase A), and found that both Aldoc and Bnip3 were induced in Vhl^cKO^ thymi relative to controls (Figure 3C). These results indicate that Vhl deficiency in TECs results in Hif1a protein accumulation and the up-regulation of a specific subset of Hif1a target genes.

### Metabolic shifts in Vhl-deficient TECs

In humans, heritable Vhl mutations result in von Hippel-Lindau disease,^33^ a multi-system disorder in which patients have a dramatically increased risk for a range of tumor types.^34^ Clear cell renal cell carcinomas (ccRCCs) are a frequent complication observed in von Hippel-Lindau disease, and one of the defining histological attributes of this tumor type is the storage of large quantities of glycogen.^35^ The loss of Vhl has been linked to glycogen accumulation in other cell types such as hepatocytes,^36^^,^^37^ suggesting a glycogenic phenotype is frequently associated with Vhl deficiency. We therefore used periodic-acid-Schiff (PAS) staining to determine if glycogen storage was increased in Vhl^cKO^ thymi. Sections from E15.5 control thymi revealed little PAS reactivity, with staining largely restricted to fine granules located in the cortical region of the thymic lobes (Figures 3D and S3A). In contrast, extensive PAS staining was observed throughout the thymic lobes of Vhl^cKO^ embryos, and large PAS^+^ granules were frequently observed in these sections. PAS-diastase staining (PAS-D)^38^ confirmed that the PAS^+^ material accumulating in Vhl^cKO^ thymi was glycogen (Figures 3D and S3B).

Several of the enzymes involved in glycogen synthesis, including Ppp1rc3 (also known as protein-targeted to glycogen, PTG^39^), and glycogen-synthase 1 (Gys1^40^) are known Hif1a target genes, and qPCR revealed that both of these genes were up-regulated in Vhl^cKO^ thymi (Figure 3E). ISH for two representative metabolic genes, Aldoc and Gys1, confirmed the up-regulation of these Hif1a target genes in Vhl^cKO^ thymi (Figure 3F). Collectively these results indicate that the loss of Vhl in TECs causes a shift in TEC metabolism that results in the substantial accumulation of glycogen in the thymus.

### Hif1a expression is dispensable for TEC differentiation

**Figure 4:**
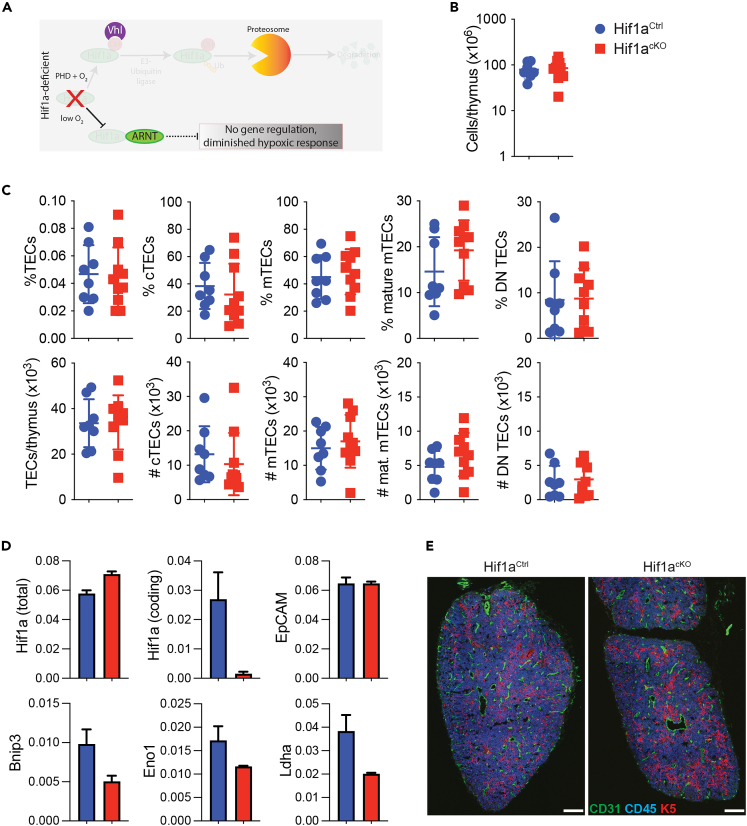
Hif1a is not required for TEC development and function A schematic indicating the consequences of Hif1a deficiency is shown in (A); in this setting the canonical hypoxic response induced by Hif1a-ARNT heterodimers is lost. Thymi were collected from Hif1a^Ctrl^ and Hif1a^cKO^ mice aged 12 weeks and analyzed by flow cytometry. Total cell counts (B), as well as the proportions (C, top row) and numbers (C, bottom row) of TECs, cTECs (UEA-1^-^Ly51^+^), mTECs (UEA-1^+^Ly51^-^), mature mTECs (MHC2^hi^CD80^+^), and double-negative TECs (UEA-1^-^Ly51^-^) were determined. Each data point represents an individual mouse (Hif1a^Ctrl^ in blue, *n* = 8; Hif1a^cKO^ in red, *n* = 10), mean ± SD is indicated. (D) qPCR was used to evaluate expression of the indicated genes by TECs sorted from E17.5 Hif1a^Ctrl^ (blue, *n* = 4) and Hif1a^cKO^ embryos (red, *n* = 3); mean ± SEM are indicated. For Hif1a two primer pairs were used; Hif1a (total) indicates a primer pair that detects Hif1a transcripts arising from all configurations of the Hif1a allele (WT, flox and Δ), while the Hif1a (coding) primer pair detects only coding transcripts (those derived from WT and un-recombined floxed-alleles), and not Δ-derived transcripts, as one of the primers binds within the floxed Hif1a coding exon deleted by Cre. Immunofluorescent staining of E17.5 thymi (E) revealed no structural defects in Hif1a^cKO^ embryos (scale bars, 100 μm, results are representative of 3 embryos per genotype).

The aforementioned results demonstrate that, in the absence of Vhl, Hif1a protein accumulates in TECs and that this accumulation correlates with the induction of several known Hif1a targets. We therefore decided to test if Hif1a up-regulation was the cause of the TEC defects observed in Vhl^cKO^ thymi. Since Hif1a deficiency (see schematic in Figure 4A) is embryonic lethal,^41^^,^^42^ we generated Hif1a conditional knockout mice (Hif1a^cKO^ mice, Hif1a^fl/fl^; Foxn1-Cre^+^) and compared them with littermate controls (Hif1a^Ctrl^, Hif1a^+/fl^; Foxn1-Cre^+^). Mice lacking Hif1a expression in the skin are viable^43^^,^^44^; therefore we were able to analyze the thymus phenotype of 12-week-old Hif1a^cKO^ mice. Adult Hif1a^cKO^ mice exhibited normal numbers of thymocytes (Figure 4B), and detailed examination of TEC subsets, including cTECs (Ly51^+^UEA-1^-^), mTECs (Ly51^−^UEA-1^+^), and mature mTECs (MHC2^hi^CD80^+^), failed to reveal any disruption in TEC numbers or differentiation (Figure 4C). Embryonic thymus development also appears normal in these mice. We used qPCR on sorted Hif1a^cKO^ TECs to confirm that Hif1a was efficiently deleted in our model (Figure 4D). Although EpCAM expression was similar between Hif1a^Ctrl^ and Hif1a^cKO^ TECs, we found that the expression of some known Hif1a target genes, including Bnip3, Eno1 and Ldha, was decreased in Hif1a^cKO^ TECs, but not entirely lost (Figure 4D). Despite these reductions, immunofluorescent staining of E18.5 thymi demonstrated that Hif1a^cKO^ thymi had a normal thymic architecture (Figure 4E). These results revealed that although the thymus is a low-oxygen environment, TEC-specific Hif1a expression is dispensable for thymus development and homeostasis.

### Rescue of TEC defects by combined Vhl-Hif1a deficiency

**Figure 5:**
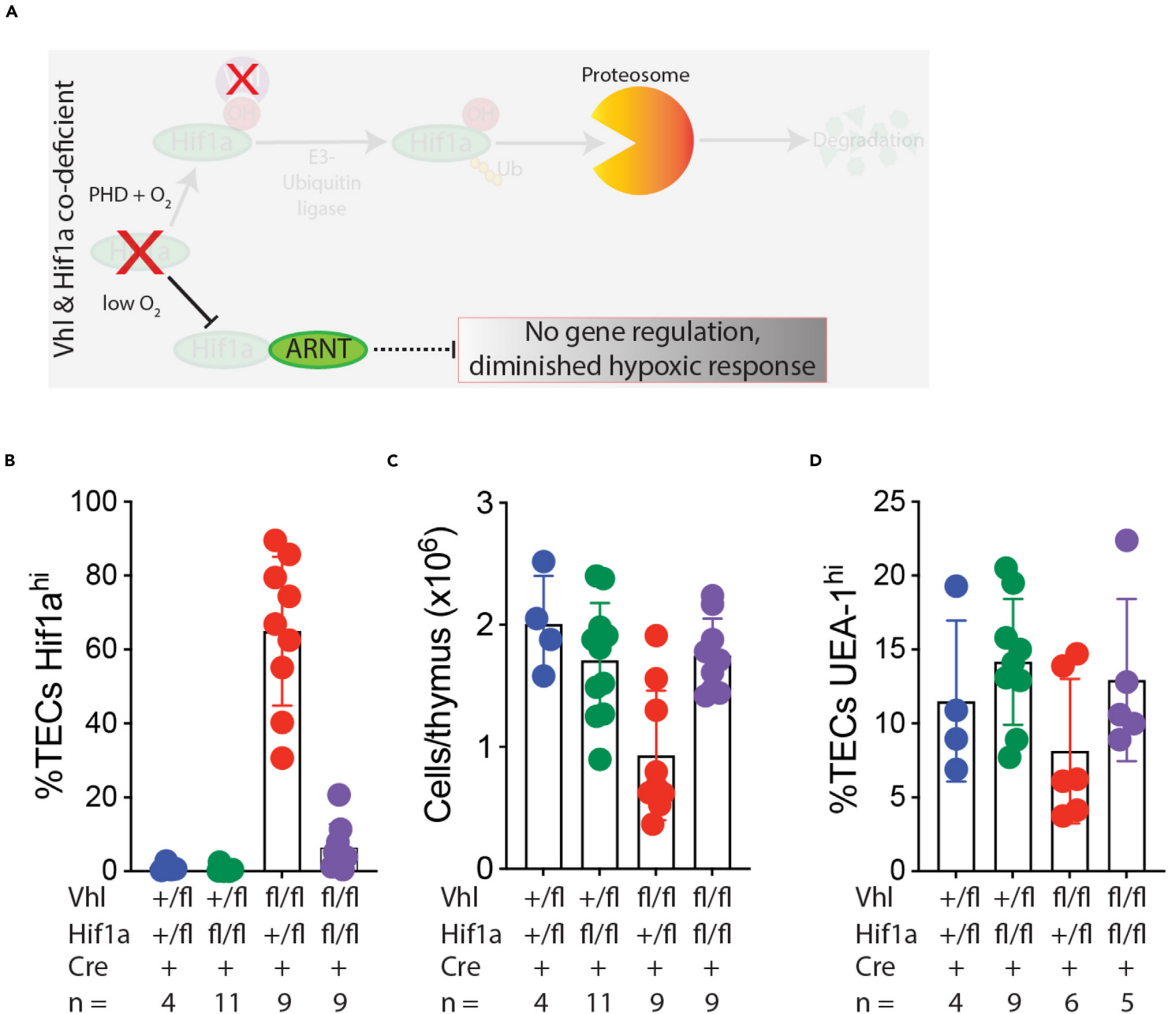
Hif1a co-depletion rescues mTEC development and thymopoiesis in Vhl-deficient thymi A schematic indicating the outcome of combined Hif1a and Vhl deficiency is depicted in (A); co-ablation of Hif1a is expected to prevent the Hif1a-dependent hypoxic response that is typically induced as a consequence of Vhl deficiency. The results of the genetic test combining Hif1a and Vhl deficiency in TECs are shown in (B and C). The proportion of TECs with a Hif1a^hi^ phenotype (B), together with the total number of cells (C), and the proportion of UEA-1^+^ TECs (D) found in thymi isolated from E17.5 embryos of the indicated genotypes are shown. The number of embryos analyzed per genotype is indicated below each column. Each data point represents an individual embryo, columns indicate the mean ± SD for each group.

Given that Hif1a is over-expressed in Vhl^cKO^ TECs, but Hif1a is dispensable for TEC development, we next sought to determine if the defects observed in Vhl^cKO^ thymi could be rescued by co-ablation of Hif1a (see schematic in Figures 5A and S4D). We therefore generated conditional knockout mice lacking Vhl and Hif1a in various combinations. We used Vhl^+/fl^; Hif1a^+/fl^; Foxn-Cre^+^ mice as controls and compared them to Hif1a-deficient mice (Vhl^+/fl^; Hif1a^fl/fl^; Foxn1-Cre^+^), Vhl-deficient mice lacking a single allele of Hif1a (Vhl^fl/fl^; Hif1a^+/fl^; Foxn1-Cre^+^), and mice deficient for both Vhl and Hif1a (Vhl^fl/fl^; Hif1a^fl/fl^; Foxn1-Cre^+^). This genetic test revealed that the co-ablation of both Hif1a alleles was necessary to prevent the accumulation of Hif1a^hi^ TECs in E17.5 Vhl-deficient thymi (Figure 5B) and that complete co-ablation of Hif1a could rescue thymocyte numbers in Vhl-deficient embryos (Figure 5C). Co-ablation of one allele of Hif1a was sufficient to at least partially restore mTEC development in E17.5 Vhl-deficient embryos, and Vhl-deficient embryos in which both Hif1a alleles were deleted had normal proportions of UEA-1^+^ mTECs (Figure 5D).

**Figure 6:**
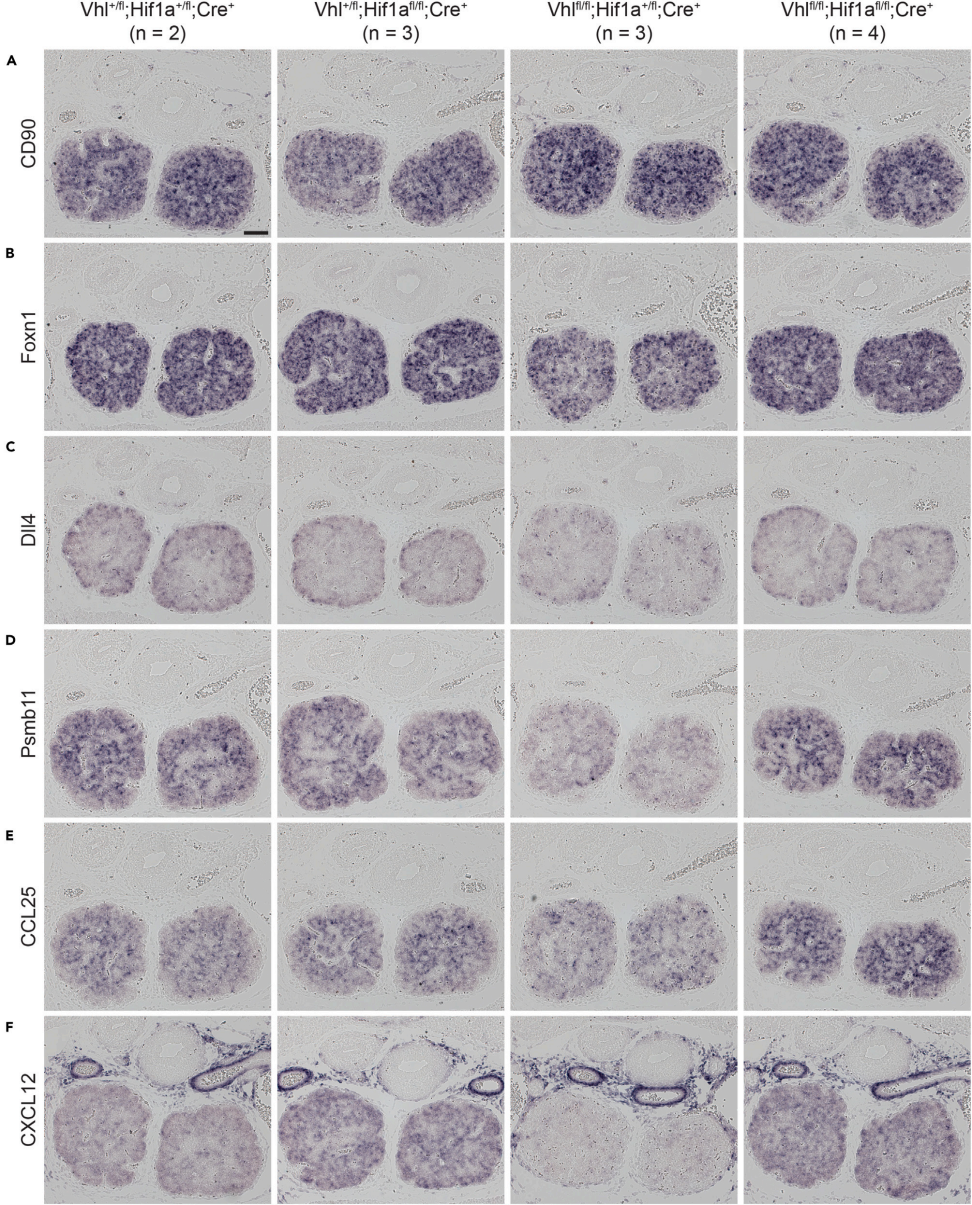
Hif1a co-depletion rescues gene expression in Vhl-deficient thymi Sections from E15.5 thymi were subjected to ISH analysis using probes for CD90 (A), Foxn1 (B), Dll4 (C), Psmb11 (D), CCL25 (E), and CXCL12 (F). Embryo genotypes and the number of embryos examined are indicated at the top of each column. The scale bar in the first panel of (A) indicates 100 μm and applies to all panels in the figure.

Surprisingly, deletion of a single allele of Hif1a was sufficient to revert many of the defects observed in Vhl^cKO^ thymi. Examination of E15.5 embryos revealed that loss of one or both Hif1a alleles was sufficient to restore the recruitment of CD90^+^ cells to Vhl-deficient thymic lobes (Figure 6A). Foxn1 expression, which is substantially decreased in Vhl^cKO^ embryos, could be restored by co-depletion of Hif1a (Figure 6B), and consistent with this the expression of several Foxn1 downstream genes, including Dll4 (Figure 6C), Psmb11 (Figure 6D), CCL25 (Figure 6E), and CXCL12 (Figure 6F), was also restored by Hif1a co-depletion. In the case of Foxn1, Psmb11, and CXCL12, staining was slightly weaker in Vhl-deficient embryos in which only one Hif1a allele was ablated (i.e., Vhl-knockout, Hif1a-haploinsufficient) compared to double knockouts or controls, suggesting an intermediate rescue in this setting.

**Figure 7:**
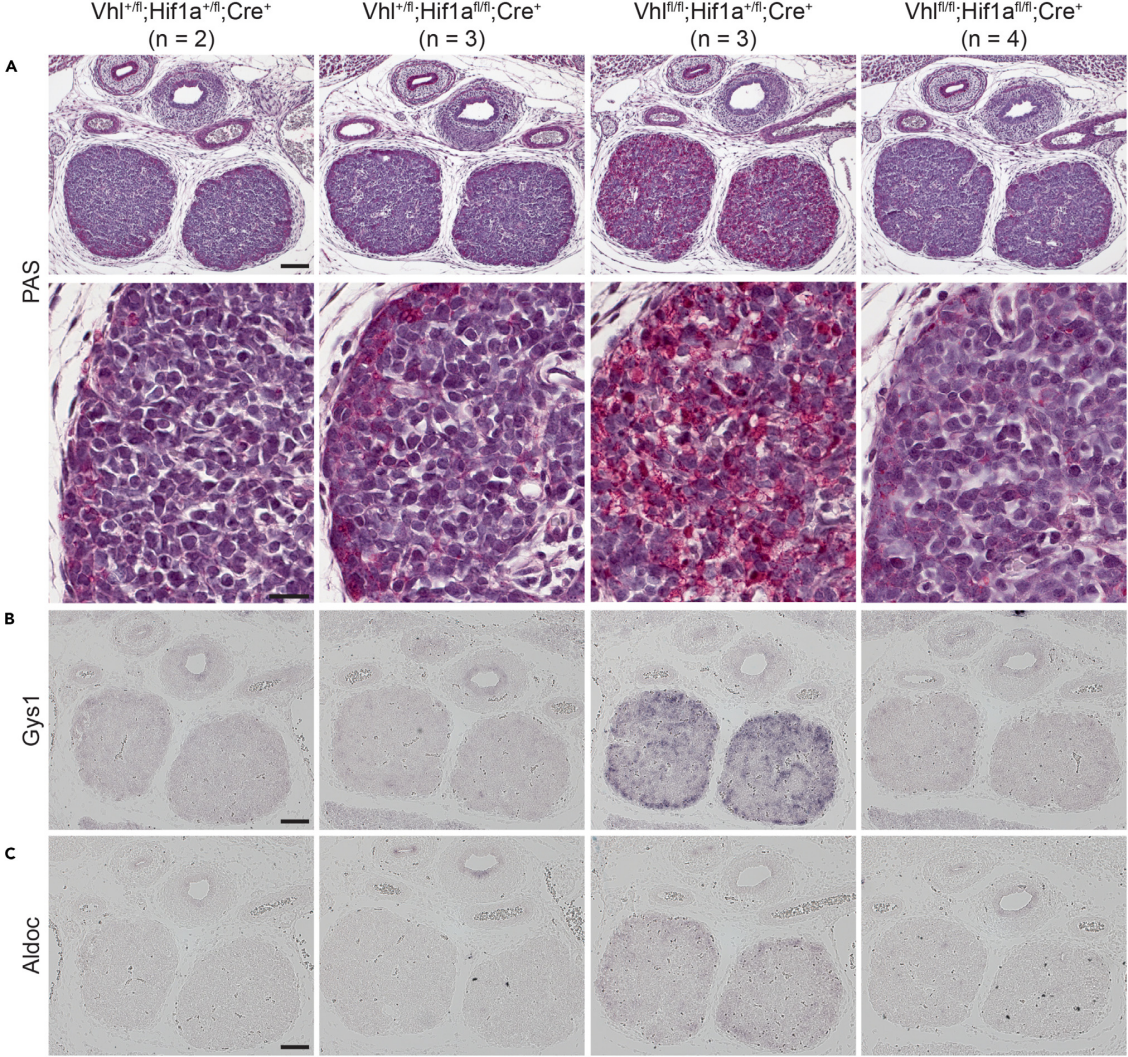
Hif1a co-depletion rescues metabolic defects in Vhl-deficient thymi Sections from E15.5 embryos of the indicated genotypes were subjected to PAS staining (A) or ISH (B and C). (A) Panoramic (top row, scale bar, 100 μm) and high-magnification (bottom row, scale bar, 20 μm) views of PAS-stained thymus sections. ISH with probes for Gys1 or Aldoc is depicted in (B and C), respectively (scale bars, 100 μm). The scale bars depicted apply to all images within the same row.

As Hif1a co-depletion was sufficient to restore the expression of many genes required for thymopoiesis in Vhl-deficient TECs, we then asked if the expression of metabolic genes was also normalized in double-deficient mice. PAS staining revealed that the glycogen accumulation phenotype observed in Vhl-deficient TECs could be reverted by co-ablation of Hif1a expression (Figure 7A). Interestingly, however, reversion of the glycogen accumulation phenotype required that both alleles of Hif1a were removed, as Vhl-deficient, Hif1a-haploinsufficient thymi still displayed extensive PAS staining. This observation correlated well with Gys1 expression: ISH analysis revealed that Gys1 was still elevated in Vhl^fl/fl^; Hif1a^+/fl^; Foxn1-Cre^+^ embryos but was undetectable in Vhl^fl/fl^; Hif1a^fl/fl^; Foxn1-Cre^+^ thymi (Figure 7B). Aldoc overexpression in Vhl-deficient thymi was also reverted by co-depletion of Hif1a, with intermediate levels observed in Vhl-deficient, Hif1a-haploinsufficient embryos (Figure 7C). Collectively, these results demonstrate that the glycogen accumulation phenotype induced by Vhl loss is Hif1a dependent. Interestingly, however, although Vhl-deficient, Hif1a-haploinsufficient thymi exhibited extensive glycogen accumulation, these thymi were nevertheless partially repopulated with thymocytes, indicating that the glycogenic phenotype is not the only factor that impedes thymopoiesis in Vhl^cKO^ embryos. Collectively, these results indicate that, although Vhl can potentially regulate a number of targets,^45^^,^^46^ its primary function in TECs is to constrain unwanted Hif1a activity.

## Discussion

Our data demonstrate that sustained Hif1a overexpression conflicts with TEC-specific functions and that Vhl is required to constrain Hif1a function, which would otherwise impede TEC differentiation and the production of T cells. These results imply that TECs are instead reliant on Hif1a-independent pathways in order to cope with low-oxygen conditions in the thymus, while simultaneously performing their essential thymopoietic functions. Interestingly, a number of other transcription factors^47^ and epigenetic regulators^48^^,^^49^ have been reported to modulate gene expression in response to hypoxia, and several of these, such as nuclear factor κB (NFκB),^50^ c-Myc.^51^^,^^52^ and p53,^53^ are known to be important for TEC function. Future investigation of these HIF-independent pathways in the thymus may help to reveal how TECs cope with their hypoxic environment.

In this study we have focused on the role of the Vhl–Hif1a axis exclusively in TECs; however, thymocytes also develop in low-oxygen thymic microenvironments. Interestingly, thymocyte numbers are reportedly normal in mice specifically lacking Hif1a in T cells^54^; however deletion of Vhl has been reported to significantly impede thymocyte differentiation.^55^ These observations parallel the phenotypes we observe for TECs and indicate that both TECs and thymocytes adapt to the low-oxygen thymic microenvironment via Hif1a-independent mechanisms.

With regard to the role of Hif1a in the thymus, it is important to specify that in this study we focused primarily on the embryonic phase of thymus development, under steady-state conditions. Examination of thymus tissue isolated from pediatric congenital heart disease patients has demonstrated that Hif1a expression is low in thymi from acyanotic patients (consistent with the low/absent Hif1a expression we observe in mouse TECs) but strongly induced in the thymus of cyanotic patients.^56^ This suggests that Hif1a may have a role to play in situations when thymic oxygen levels drop below the normal state of physiological hypoxia, into a pathologically hypoxic range. Exposure of mice to hypoxic conditions is known to cause rapid thymus involution, which is reverted upon return to normoxia.^57^ Therefore, although TEC-expressed Hif1a is not essential for thymus function during normal development, our results do not preclude a function for Hif1a in TECs under stress conditions.

It may be noted that in our study we have focused on embryonic thymus development, while the reported measurements of thymic oxygen levels were taken in adult mice. Direct measurement of pO_2_ in embryonic thymic lobes is a technically challenging proposition and has not been performed to our knowledge. Nevertheless, several lines of evidence indicate that the embryonic thymus develops under low-oxygen conditions. In mammals, the early stages of embryogenesis are known to occur under low-oxygen conditions,^58^ and the entire process of normal fetal development takes place under conditions of relative hypoxia: fetal arterial pO_2_ is normally in the range of 20–30 mmHg, compared to ≈100 mmHg in adults.^59^ Furthermore, many of the steps in thymus organogenesis occur prior to the establishment of maternal arterial blood flow to the placenta, a key event required for efficient gas and nutrient exchange between the mother and embryo. In human embryos, Foxn1 expression first becomes detectable during the sixth week of gestation, colonization of the thymic rudiment by hematopoietic cells occurs around week 7, and the first T-committed progenitors are observed at around week 8.^60^ These key events in thymus organogenesis therefore occur within the hypoxic uterine environment,^61^ at a time before significant maternal arterial circulation is established within the placenta.^62^

One limitation of our study is that we have focused on the mid- to late-gestation phase of thymus development. Follow-up studies on the role of Vhl and Hif1a in thymus biology at other stages, such as at the very inception of thymus development from the third pharyngeal pouch, or in adult mice, may reveal addition insights into how oxygen and oxygen-sensitive pathway regulate thymus biology.

On face value, it would seem that there is an apparent discord regarding the role of oxygen in the thymus in *in vivo* versus *in vitro* settings. Although oxygen levels are low *in vivo*, several studies have clearly demonstrated that oxygen availability plays a key role in fetal thymi organ culture (FTOC). Early studies on the feasibility of FTOC showed that oxygen availability was a limiting factor for viable cultures,^63^ and the use of air-liquid interface cultures to maximize gas exchange has become the most prevalent FTOC technique, and a commonly used model for studying T cell development *in vitro.*^64^^,^^65^ The importance of oxygen as a key factor in FTOC is further supported by studies demonstrating that the successful *in vitro* repopulation of deoxyguanosine-treated thymic lobes requires high oxygen pressure^66^ and that oxygen levels play an important role in maintaining both Foxn1 and MHC2 levels in FTOCs, including submersion cultures.^67^ Although high-oxygen cultures are therefore advantageous for T cell development in FTOCs, it remains difficult to assess the true availability of oxygen at the cellular level within the cultured thymic lobes: local oxygen pressure and ambient oxygen pressure can differ dramatically due to factors such as diffusion distances, consumption rates, and solubility, among other considerations.^68^^,^^69^ Therefore, it is quite possible that the high ambient O_2_ levels used in FTOCs are needed to prevent complete anoxia within the lobes, rather than to generate a highly oxygenated microenvironment at a cellular level, which would likely be poorly representative of the physiological situation.^70^

Our results emphasize that oxygen-sensitive pathways can have a dramatic influence on thymus biology and suggest that attempting to more closely mimic the physiology of the thymus could help to optimize *in vitro* culture systems for both experimental and clinical applications. For example, incubating cultures at “physoxia” (defined as a 5% O_2_ ambient environment, as opposed to the 21% O_2_ that is typically used) has been shown to enhance T cell differentiation in artificial thymic organoid (ATO) cultures.^71^ Approaches for thymic organ culture are constantly evolving,^72^^,^^73^ and it would seem that the incorporation of novel techniques to more precisely regulate oxygen availability within critical microenvironments^74^ could significantly enhance their efficacy in the future. Such advances may serve to improve the generation of T cells for adoptive immunotherapy,^72^ or to enhance the use of cultured thymic tissue in transplantation to treat athymia.^75^^,^^76^

### Limitations of the study

The role of Vhl was tested only during embryonic development, and not yet in adult mice. Additionally, although we found no obvious function for Hif1a in TECs during development, and under steady-state conditions, it remains possible that this pathway could still play a role under stress conditions, or following damage to the thymic epithelium.

## STAR★Methods

### Key resources table

**Table:** 

REAGENT or RESOURCE	SOURCE	IDENTIFIER
**Antibodies**		

anti-Aire, Alexa 488	eBioscience	5H12; RRID:AB_10852560
anti-CD3e, biotin	eBioscience	145-2C11; RRID:AB_466319
anti-CD16/32, unconjugated	eBioscience	93; RRID:AB_467134
anti-CD31, biotin	BD Pharmingen	MEC 13.3; RRID:AB_394817
anti-CD45, PE-Cy7	BioLegend	30-F11; RRID:AB_312978
anti-CD45, Alexa 488	BioLegend	30-F11; RRID:AB_493532
anti-CD45, Alexa 647	BioLegend	30-F11 RRID:AB_493534
anti-CD80, biotin	BioLegend	16-10A1; RRID:AB_313125
anti-EpCAM, APC	BioLegend	G8.8; RRID:AB_1134102
anti-ERTR7, unconjugated	Origene	ERTR7; Cat#BM4018
anti-Hif1a, PE	Cell Signaling Technology	D1S7W, RRID:AB_2799565
anti-Keratin-5, unconjugated	BioLegend	polyclonal; RRID:AB_2565050
anti-Keratin-8, unconjugated	Kemler, R et al.^79^	TROMA-1
anti-Keratin-18, biotin	PROGEN	Ks18.04; Cat#61528
anti-Ki67, FITC	eBioscience	SolA15; RRID:AB_11151330
anti-Ly51, PE	eBioscience	6C3; RRID:AB_466016
anti-MHC1, A488	BioLegend	AF6-88.5; RRID:AB_492915
anti-MHC2, PE	BioLegend	M5/114.15.2; RRID:AB_313323
anti-Psmb11, unconjugated	MBL International	polyclonal; RRID:AB_2171885
anti-Rabbit IgG, Alexa555	Invitrogen	polyclonal; RRID:AB_2535849
anti-Rat IgG, Alexa647	Molecular probes	polyclonal; RRID:AB_141778

**Chemicals, peptides, and recombinant proteins**		

UEA-1, FITC	Vector Laboratories	RRID:AB_2336767
Streptavidin, Alexa647	Invitrogen	Cat#S32357
Streptavidin, Cy3	Jackson ImmunoResearch	RRID:AB_2337244
Streptavidin, Alexa488	Jackson ImmunoResearch	RRID:AB_2337249
collagenase type 4	Worthington Biochemical Corporation	Cat#LS004189
neutral protease (dispase)	Worthington Biochemical Corporation	Cat#LS02104
DNaseI	Roche	Cat#11284932001
EDTA	Carl Roth	Cat#8043.1
T7 RNA polymerase	Roche	Cat#10881767001
SP6 RNA polymerase	Roche	Cat#10810274001
Superscript IV Reverse Transcriptase	Invitrogen	Cat#18090050
FastStart Universal Probe Master (ROX) mix	Roche	Cat#4913949001
Fixation/Permeabilization Buffer	eBioscience	Cat#00-5123-43
Permeabilization Buffer	eBioscience	Cat#00-8333-56
TRI Reagent	Sigma-Aldrich	Cat#T9424
hydroxystilbamidine	Enzo Life Sciences GmbH	Cat#ENZ-52253
DAPI	Roche	Cat#10236276001
alpha-amylase type VI-B	Sigma-Aldrich	Cat#A3176

**Critical commercial assays**		

DIG RNA labelling Mix	Roche	Cat#11277073910
Periodic Acid-Schiff Staining Kit	Sigma-Aldrich	Cat#395B-1KT
Endogenous Biotin-Blocking Kit	Invitrogen	Cat#E21390

**Experimental models: Organisms/strains**		

Mouse: Vhl^tm1Jae^ (Vhl-flox)	The Jackson Laboratory	RRID:IMSR_JAX:004081
Mouse: Hif1a^tm3Rsjo^ (Hif1a-flox)	The Jackson Laboratory	RRID:IMSR_JAX:007561
Mouse: Tg(Foxn1-cre)1Tbo	Soza-Ried C et al.^21^	N/A

**Oligonucleotides**		

ISH probe sequences are listed in Table S1.	This paper	N/A
QPCR Primer sequences are listed in Table S3.	This paper	N/A

**Software and algorithms**		

BD FACSDiva	BD Life Sciences	version 8.0.1
FlowJo	FlowJo	version 10.7.1
Axiovision	Carl Zeiss	version Rel4.8.2
Zeiss ZEN	Carl Zeiss	version 3.2
Prism	GraphPad	version 9.3.1

### Resource availability

#### Lead contact

Further information and requests for resources and reagents should be directed to, and will be fulfilled by, the lead contact, Jeremy Swann (swann@ie-freiburg.mpg.de).

#### Materials availability

This study did not generate new unique reagents.

#### Data and code availability

• All data reported in this paper will be shared by the lead contact (swann@ie-freiburg.mpg.de) upon request.
• This paper does not report original code.
• Any additional information required to reanalyze the data reported in this paper is available from the lead contact (swann@ie-freiburg.mpg.de) upon request.

### Experimental model and study participant details

#### Mice

Foxn1-Cre,^21^ Hif1a^77^ and Vhl^20^ mice have all been previously described, and were maintained on a predominantly C57BL6/J background. Mice were housed under conventional conditions in individually ventilated cages at the Max Planck Institute of Immunobiology and Epigenetics animal facility. Regular health monitoring was performed according to FELASA guidelines,^78^ and all mice were healthy at the time of use. Mice and embryos were used at various ages, which are specified in the text and each figure legend. Mice and embryos used in this study were not subjected to any prior treatments or previous experimental procedures. Mice and embryos of both sexes were used, and no significant effect of sex was noted. For timed matings, the day of plug detection was designated as E0.5. All animal experiments were performed in accordance with relevant guidelines and regulations, approved by the review committee of the Max Planck Institute of Immunobiology and Epigenetics and the Regierungspräsidium Freiburg, Germany (license Az 35-9185.81/G-12/85).

### Method details

#### Histology

Embryos for H&E staining and ISH were fixed in 4% PFA at 4°C for 48hrs, and subsequently embedded in paraffin using standard techniques. PAS staining was performed using the Periodic Acid-Schiff Staining System (Sigma-Aldrich, 395B). In the case of PAS-D staining, sections were pre-treated with a 0.5% solution of α-amylase type VI-B (Sigma-Aldrich) for 20 min at room temperature. All embryo sections were cut in the transverse plane, dorsal is up, ventral is down in all figures.

#### RNA *in situ* hybridization

Digoxigenin-labeled riboprobes were generated by *in vitro* transcription utilising T7 or SP6 RNA polymerases and DIG RNA labelling Mix (all from Roche). RNA *in situ* hybridisation (ISH) on paraffin sections was performed using DIG-labelled probes as described in.^79^ Sequences details for the various probes used are given in Table S1. Images were acquired using a Zeiss ImagerZ.1 microscope equipped with a Zeiss AxioCam 305 color camera and Zeiss ZEN 3.2 software.

#### Immunofluorescence

E15.5 embryos for IF were embedded directly in Tissue-Tek O.C.T. Compound and frozen on dry ice. E17.5 thymi were harvested, fixed for 15 min in 4% PFA at 4°C, washed in PBS, then infiltrated with 16% sucrose for 3 h (all at 4°C), before embedding in O.C.T. Cryoblocks were sectioned on a Leica CM3050S cryostat at 8-10μm thickness. For immunofluorescent staining, sections were first blocked with mouse IgG (Jax Immunoresearch) diluted 1:50 in PBS/Tween (PBS containing 0.5% BSA and 0.2% Tween 20) for 30 min, washed, then incubated with Endogenous Biotin Block (Invitrogen) for a further 30 min. After blocking, sections were washed and incubated for 1 h with primary antibodies diluted in PBS/Tween supplemented with either 5% normal goat serum or 5% normal rat serum. Sections were then washed and incubated for 30 min with secondary antibodies/streptavidin diluted in PBS/Tween supplemented with either 5% normal goat serum or 5% normal rat serum. After staining sections were washed, stained with DAPI (50 ng/mL in PBS), washed again, and finally mounted in Fluormount G (Invitrogen). All washing steps were performed with PBS, and the entire staining procedure was performed at room temperature. Images were acquired on a Zeiss ImagerZ.1 microscope equipped with a Zeiss AxioCam MRm camera and Axiovision Rel4.8.2 software. Details for all antibodies used for immunofluorescence can be found in Table S2. UEA-1 staining was performed in parallel with antibody staining, UEA-1-biotin (Vector Laboratories, B-1065) was included in the primary stain, and detected with streptavidin-A647 (Invitrogen, S32357) in the secondary step.

#### Flow cytometry

To generate single cell suspensions for TEC analysis thymi were minced with scissors, and then digested with a cocktail of collagenase type 4 (200 μg/mL), neutral protease (200 μg/mL) and DNaseI (500 ng/mL) in RPMI 1640 + 2% FCS for up to 90 min at 37°C with agitation. Following digestion, EDTA was added to a final concentration of 2mM, and samples were incubated for a further 5 min to disaggregate any remaining cell clumps. Cells were then washed and resuspended in RPMI 1640 + 2% FCS for counting. Cell numbers were determined using a CASY counter (Innovatis). Cells were transferred to 96-well round bottom plates and re-suspended in 0.5% BSA and 0.02% NaN_3_ (PBS/BSA) for surface staining. Non-specific antibody binding was blocked by the addition of unlabeled anti-CD16/32 antibodies to the staining solution. After surface staining, cells were either washed and resuspended in PBS/BSA containing 1 μg/mL of the viability dye hydroxystilbamidine (Enzo Life Sciences GmbH) for analysis, or further processed for intracellular staining. Intracellular staining for Hif1a was performed using the eBioscience Fixation/Permeabilization (00-5123-43) and Permeabilization (00-8333-56) buffers diluted as per the manufacturer’s instructions. Surface-stained cells were fixed with Fixation/Permeabilization solution for 20 min on ice, centrifuged for 5 min at 600 x g, then washed twice with 200μL Permeabilization buffer. Fixed and permeabilized cells were then resuspended in 50μL Permeabilization buffer containing the anti-Hif1a-PE antibody diluted 1:200, and incubated at 4°C for 30 min with gentle shaking. Stained cells were then washed twice with 200μL Permeabilization buffer, once with PBS/BSA, and finally resuspended in PBS/BSA for analysis. All washing, staining and centrifugation steps were done at 4°C, unless otherwise specified. Samples for analysis were acquired on a LSR Fortessa cytometer (Dako Cytomation-Beckman Coulter) equipped with FACSDiva software. Data was subsequently analysed using FlowJo 10.7.1. Details for all antibodies used for flow cytometry can be found in Table S2. UEA-1-FITC (Vector Laboratories, FL-1061) was additionally used for identification of mTEC by flow cytometry, and added in parallel with antibodies for surface staining. All antibodies were purchased from commercial suppliers, with the exception of the anti-keratin-8 antibody, which was purified in from the hybridoma TROMA-1.^80^

#### Cell sorting

Cell suspensions were prepared as described above, and stained with CD45 and EpCAM antibodies diluted in RPMI 1640 + 2% FCS for 30 min, washed once, and then sorted on using a MoFlo cell sorter (Dako Cytomation-Beckman Coulter). CD45^−^EpCAM^+^ TECs were sorted directly into TRI Reagent (Sigma-Aldrich) to preserve RNA for analysis.

#### RNA extraction and QPCR

RNA was extracted from thymi or sorted TECs using the TriReagent (SigmaAldrich) extraction protocol as per the manufacturer’s instructions, and single-stranded cDNA was subsequently prepared using the Superscript IV (Invitrogen) reverse transcription system. QPCR was performed using FastStart Universal Probe Master (ROX) mix (Roche). The Applied Biosystems 7500 Fast system was used to detect the signal generated with gene-specific primers combined with 5′ FAM (6-carboxyfluorescein) labeled hydrolysis probes from either the Universal Probe Library (Roche), or custom synthesized (Eurofins Genomics) with 5′ FAM and 3′ TAMRA labels. Primer sequences and probe details are given in Table S3. Cycling conditions were: 50°C for 20 s, 95°C for 10 min, then 50 cycles of 95°C for 15 s, 60°C for 1 min. Data are presented as mean 2^−ΔΔct^ values, beta-actin was used as the reference gene.

### Quantification and statistical analysis

#### Statistical analysis

Statistical analysis was performed using GraphPad Prism, Version 9.3.1; t-tests (two-tailed) were used to determine the significance levels of the differences between the means of two independent samples. Sample sizes were estimated using G∗Power,^81^ and the number of biological replicates is indicated in each figure legend. No blinding or randomization was used.
