# Supplementary material for: Vhl safeguards thymic epithelial cell identity and thymopoietic capacity by constraining Hif1a activity during development
Source: iScience. 2024 Jun 13;27(7):110258. doi: 10.1016/j.isci.2024.110258 (PMC11261450; doi:10.1016/j.isci.2024.110258)
Supplement: Document S1. Figures S1–S4 and Tables S1–S3 [file mmc1.pdf]

**iScience, Volume *27***

## Supplemental information

# Vhl safeguards thymic epithelial cell identity and thymopoietic capacity by constraining Hif1a activity during development

**Christiane Grammer, Julia A. Komorowska, and Jeremy B. Swann**

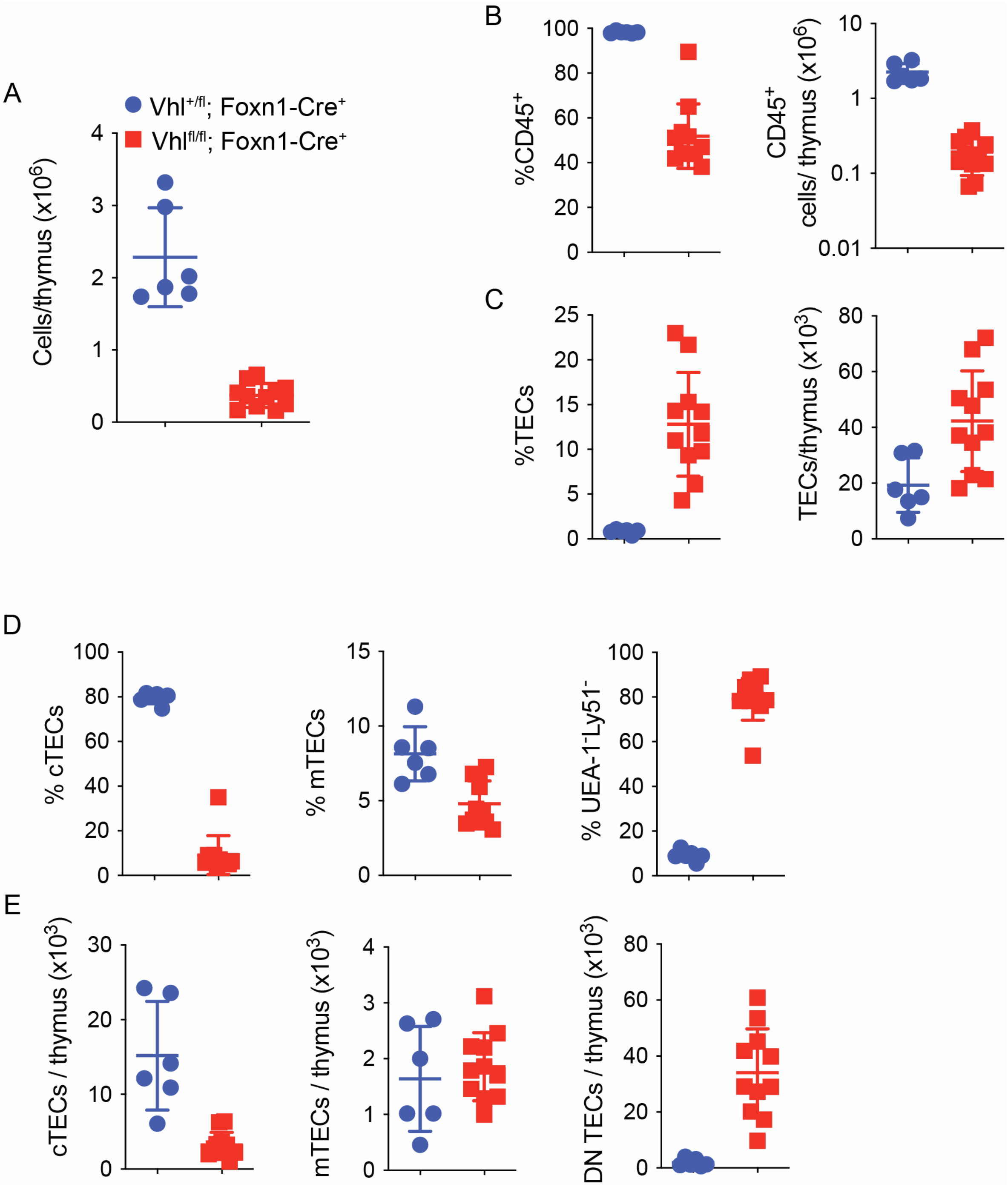


**Figure S1. Vhl-deficiency perturbs TEC differentiation and thymopoiesis (related to Figure 1).** (A) Total cell counts from thymi isolated from $Vhl^{Ctrl}$ and $Vhl^{cKO}$ E18.5 embryos. (B & C) show the proportions and total numbers of $CD45^{+}EpCAM^{-}$ hematopoietic cells (B) and $CD45^{-}EpCAM^{+}$ TECs (C), determined using the gating strategy depicted in Figure 1C. The percentages (D) and total numbers (E) of cTEC, mTEC and DN TEC subsets, as gated in Figure 1D are shown. For all plots each data point represents an individual embryo (n = 6 ($Vhl^{Ctrl}$) and 11 ($Vhl^{cKO}$)), and the mean ± SD is indicated for each genotype.

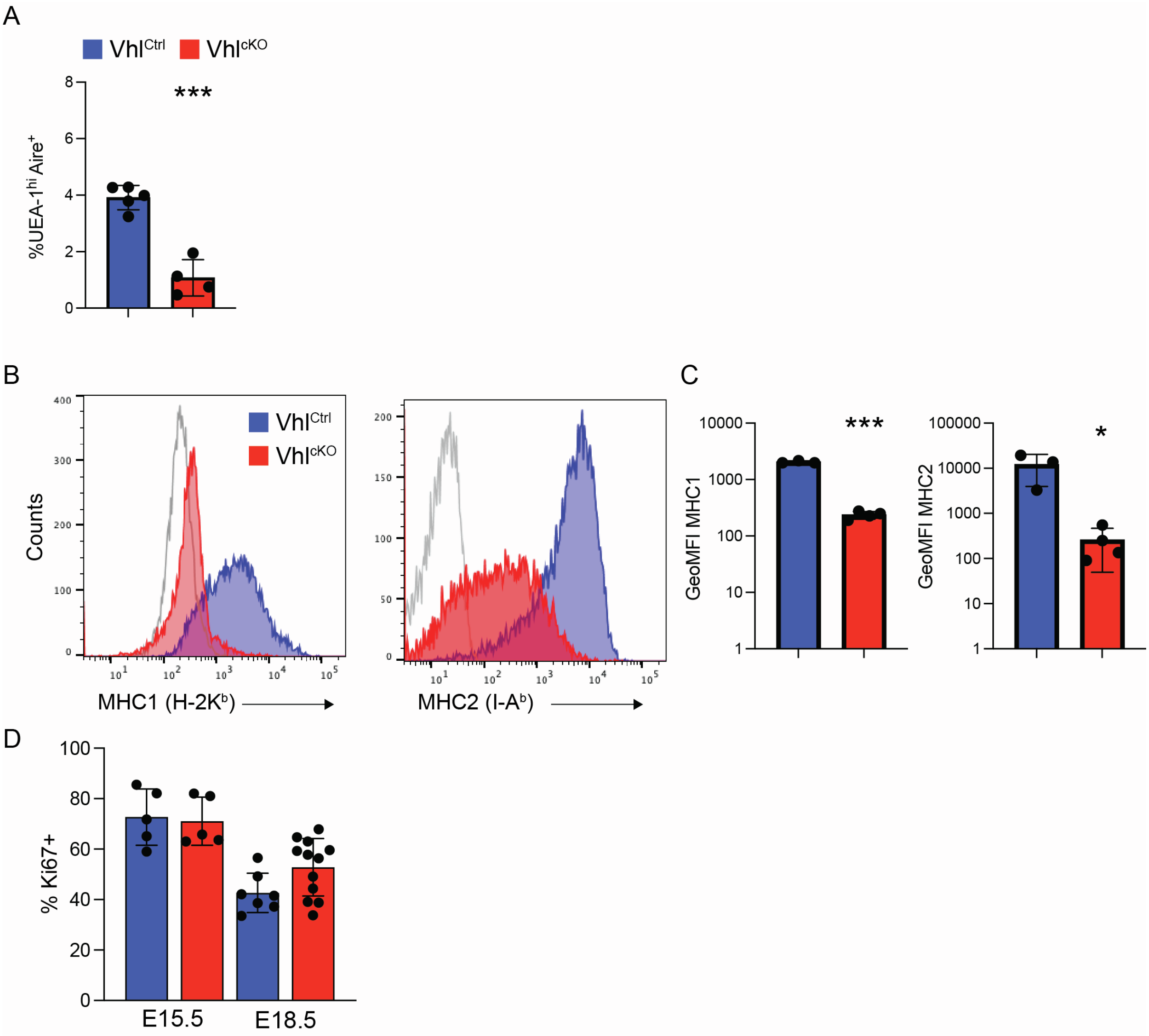


**Figure S2. Vhl-deficiency perturbs TEC differentiation, but not proliferation (related to Figure 1).**

The proportion of UEA-1$^{+}$Aire$^{+}$ mature mTECs among gated CD45$^{-}$EpCAM$^{+}$ TECs from E18.5 embryos is shown in (A). Representative staining for MHC1 and MHC2 expression by gated Vhl$^{Ctrl}$ (blue histograms), Vhl$^{cKO}$ (red histograms) E18.5 TECs is depicted in (B), with grey histograms indicating background staining with isotype controls. MHC1 and MHC2 staining is quantified in (C). The proportion of Ki67$^{+}$ TECs from at E15.5 and E18.5 embryos is shown in (D). The genotype key depicted in panel (A) applies to all graphs. Each data point in (A, C and D) represents an individual embryo, and the columns and error bars represent mean ± SD. * and *** indicate p-values ≤ 0.05 and 0.001 (two tailed t-test).

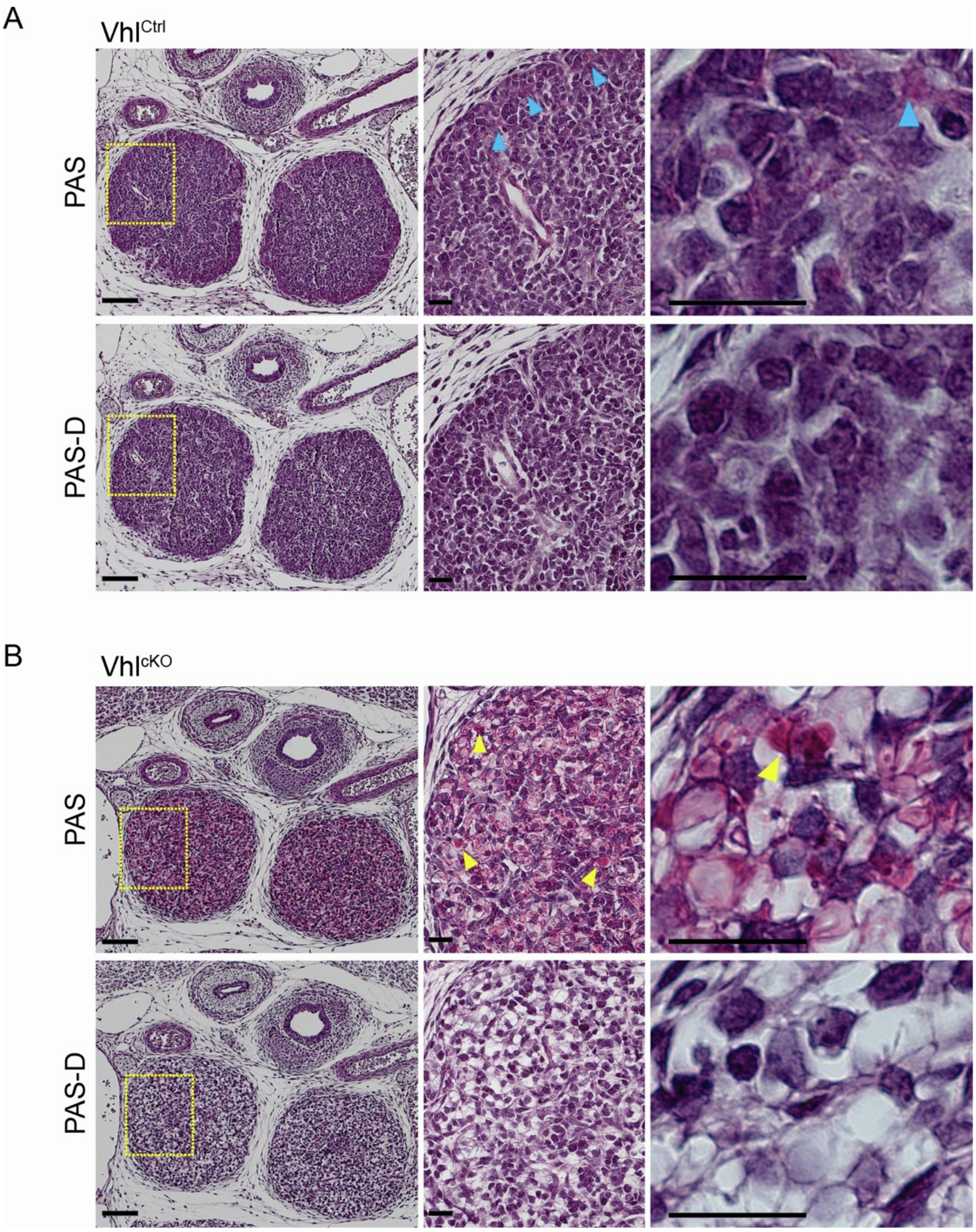


**Figure S3. PAS and PAS-D staining of embryonic thymi (related to Figure 3).**
In the PAS staining procedure, magenta staining indicates the presence of polysaccharides in tissue sections, and a hemotoxylin counterstain (which stains nuclei dark purple) is used to reveal structural context. PAS-staining of sections from E15.5 $Vhl^{Ctrl}$ (A) and $Vhl^{cKO}$ (B) embryos revealed substantial accumulation of $PAS^{+}$ material in $Vhl^{cKO}$ thymi. This staining was lost when sections were pre-treated with diastase, an enzyme which degrades glycogen (PAS-D staining protocol). For both (A) and (B) the top row depicts PAS-staining, and the bottom row PAS-D-staining of thymus sections. A panoramic overview of the thymus is shown in the first column (scale bars indicate 100μm), a zoomed in view of the regions highlighted with dashed yellow boxes is shown in the middle column (scale bars = 100μm), and a high-magnification view of the thymus is shown in the last column (scale bars = 20μm). Blue arrowheads indicate the fine $PAS^{+}$ granules observed in the cortex of $Vhl^{Ctrl}$ thymi, yellow arrowheads highlight some of the large $PAS^{+}$ granules found in $Vhl^{cKO}$ thymi. PAS-staining is lost following diastase treatment, identifying the accumulated polysaccharide as glycogen. Results are representative of at least 3 embryos examined per genotype. Note that sections in the first column of this figure are also depicted in Figure 3D, and are reproduced here to provide spatial context for the corresponding higher-magnification images.

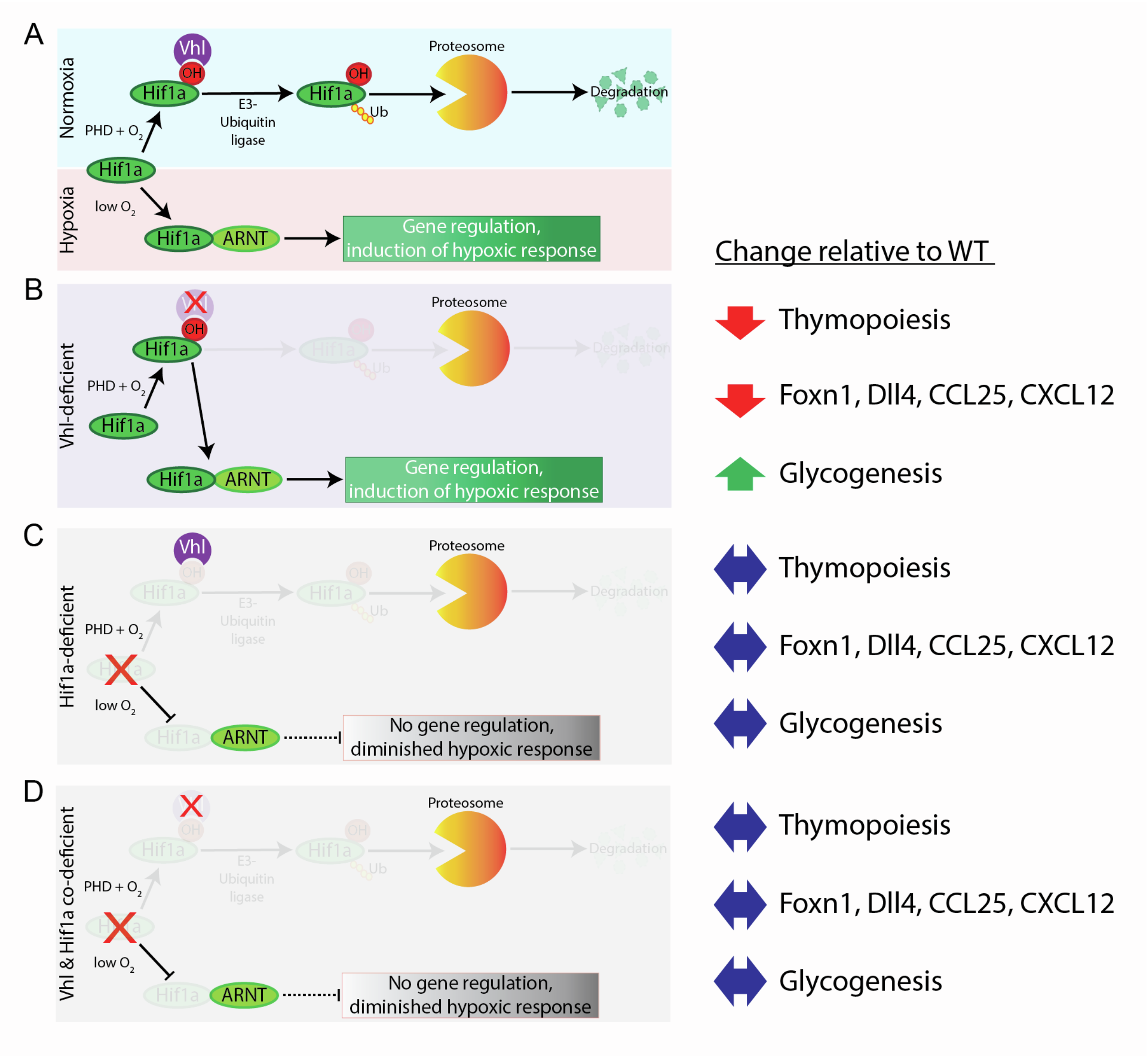


**Figure S4. Schematic of Vhl – Hif1 axis perturbations (related to Figures 1 & 5).**
In (A) the canonical fate of Hif1a protein under different oxygen conditions is depicted. When oxygen is abundant (top pathway, blue shading) PHD enzymes hydroxylate Hif1a protein, which can then be recognised by a Vhl-containing E3-ubiquitin ligase complex. Ubiquitination of Hif1a results in its rapid degradation via the proteosome. When oxygen levels are low (bottom pathway, red shading) Hif1a is no longer hydroxylated, and therefore evades the Vhl-dependent degradation pathway. Hif1a protein can then pair with its binding partner ARNT, forming a DNA-binding heterodimer that translocates to the nucleus to drive hypoxia-induced transcriptional responses. When Vhl is ablated (B), Hif1a protein is no longer targeted to the proteosome, even in the presence of oxygen. This allows Hif1a to induce a hypoxic response independent of oxygen levels. In contrast, when Hif1a is ablated (C) no hypoxic response is possible, regardless of oxygen levels; note however that Vhl is still available to service other potential target proteins. To determine if the effects of Vhl-ablation were Hif1a-dependent, we also co-ablated Vhl and Hif1a (D). In this situation no hypoxic response is possible, as is the case for Hif1a single-deficiency, however in this instance Vhl is additionally unavailable for the regulation of non-Hif1a substrates. For each genotype, a summary of the consequences of the gene perturbations on thymus biology is indicated. Red, green and blue arrows indicate an increase, decrease, or no change in the indicated parameter respectively.

**Table S1.** Probes used for RNA *in situ* hybridisation, related to STAR Methods

| Target | mRNA accession number | Probe sequence (nucleotides) |
|---|---|---|
| Aldoc | NM_001303423.1 | 477..1073 |
| CCL25 | NM_009138.3 | 119..553 |
| CD90 | NM_009382.3 | 96..617 |
| CXCL12 | NM_021704.3 | 64..479 |
| Dll4 | BC049130 | 2271..3421 |
| Foxn1 | XM_006532266.3 | 2181..3584 |
| Gys1 | NM_030678.3 | 2186..2862 |
| Psmb11 | NM_175204.4 | 411..879 |

**Table S2.** Antibodies used for flow cytometry and immunofluorescence, related to STAR Methods

| Target | Ab. Clone | Conjugate | Supplier | Product No. | Use[a] |
|---|---|---|---|---|---|
| Aire | 5H12 | Alexa488 | eBioscience | 53-5934-80 | FC |
| CD3e[b] | 145-2C11 | Biotin | eBioscience | 13-0031-82 | IF |
| CD16/32 | 93 | - | eBioscience | 14-0161-85 | FC |
| CD31[c] | MEC 13.3 | Biotin | BD Pharmingen | 553371 | IF |
| CD45 | 30-F11 | PE-Cy7 | BioLegend | 103114 | FC |
| CD45 | 30-F11 | Alexa488 | BioLegend | 103122 | IF |
| CD45 | 30-F11 | Alexa647 | BioLegend | 103124 | IF |
| CD80 | 16-10A1 | Biotin | BioLegend | 104704 | FC |
| EpCAM | G8.8 | APC | BioLegend | 118214 | FC |
| ERTR7 | ERTR7 | - | Origene | BM4018 | IF |
| Hif1a | D1S7W | PE | Cell Signaling Technology | 59370S | FC |
| Keratin-5 | polyclonal | - | BioLegend | 905501 | IF |
| Keratin-8 | TROMA-1 | - | In house | n/a | IF |
| Keratin-18[c] | Ks18.04 | Biotin | PROGEN | 61528 | IF |
| Ki67 | SolA15 | FITC | eBioscience | 11-5698-82 | FC |
| Ly51 | 6C3 | PE | eBioscience | 12-5891-83 | FC |
| MHC1 | AF6-88.5 | Alexa488 | BioLegend | 116510 | FC |
| MHC2 | M5/114.15.2 | PE | BioLegend | 107608 | FC |
| Psmb11 | polyclonal | - | MBL International | PD021 | IF |
| Rabbit IgG[d] | polyclonal | Alexa555 | Invitrogen | A21428 | IF |
| Rat IgG[e] | polyclonal | Alexa647 | Molecular probes | A21247 | IF |

[a]Immunofluorescence (IF), flow cytometry (FC)
[b]Biotin-labelled primary antibody was detected by subsequent incubation with Steptavidin-Cy3 (Jackson ImmunoResearch, 016-160-084)
[c]Biotin-labelled primary antibody was detected by subsequent incubation with Steptavidin-A488 (Jackson ImmunoResearch, 016-540-084)
[d]Secondary antibody, used to detect ERTR7-staining
[e]Secondary antibody, used to detect K5- and Psmb11-staining

**Table S3.** Primers and probes used for QPCR, related to STAR Methods

| Target | Primer 1 | Primer 2 | Probe[c] |
|---|---|---|---|
| Aldoc | tgctgccaccttatttactcc | agtggggcatgatgacagtt | UPL#6 |
| Beta-actin | ctaaggccaaccgtgaaaag | accagaggcatacagggaca | UPL#64 |
| Bnip3 | cctgtcgcagttgggttc | gaagtgcagttctacccaggag | UPL#52 |
| Eno1 | gaggcgcttagtgctgct | atagacatggcgaatttctgg | UPL#91 |
| EpCAM | agaatactgtcatttgctccaaact | gttctggatcgccccttc | UPL#52 |
| Gys1 | tgaacagcaagggttgtaagg | gtcccaaagctcacccttc | UPL#1 |
| Hif1a (total)[a] | catgatggctccctttttca | gtcacctggttgctgcaata | UPL#18 |
| Hif1a (coding)[b] | tgagcttgctcatcagttgc | tgccttcatctcatcttcactg | UPL#60 |
| Ldha | ggcactgacgcagacaag | tgatcacctcgtaggcactg | UPL#12 |
| Ppp1rc3 | aatgagctgcaccagaatg | tgaatgagccaagcaaatcc | tgctagatccacgtcct ttgacaagttcc |

[a] Primer pair detects Hif1a transcripts from WT, floxed and Δ alleles

[b] Primer pair detects Hif1a transcripts from WT and floxed, but not Δ, alleles

[c] Probes were derived from the Roche Universal Probe Library except in the case of Ppp1rc3, for which no suitable UPL probe could be identified. In this case a custom probe was synthesized, and the full probe sequence is indicated
